# Comparison of Consumption of Pulses in Two Seasons of the Year in Chile

**DOI:** 10.3390/nu15112635

**Published:** 2023-06-05

**Authors:** Claudia Barrientos-De la Rosa, Samuel Duran-Aguero, María José Mardones, Yadira Morejón, Paula García-Milla, Pablo Albornoz, Ximena Torres, Leslie Landaeta-Díaz

**Affiliations:** 1 Carrera Nutrición y Dietética, Facultad de Ciencias de la Salud, Universidad Autónoma de Chile, Providencia 7500975, Chile; claudia.barrientos@cloud.uautonoma.cl (C.B.-D.l.R.); ppaulagm@gmail.com (P.G.-M.); ximena.torres@uautonoma.cl (X.T.); 2 Escuela de Nutrición y Dietética, Facultad de Ciencias Para el Cuidado de la Salud, Universidad San Sebastián, Santiago 7511111, Chile; samuel.duran@uss.cl (S.D.-A.); maria.mardones@uss.cl (M.J.M.); 3 Centro de Investigación de Salud Pública y Epidemiología Clínica (CISPEC), Facultad de Ciencias de la Salud Eugenio Espejo (FCSEE), Universidad UTE, Quito 170150, Ecuador; ymorejon@hotmail.com; 4 Centro Integrativo de Biología y Química Aplicada (CIBQA), Universidad Bernardo O’Higgins, General Gana 1702, Santiago 8370854, Chile; 5 Fit Food Chile Company, Santiago 7500975, Chile; mtwpablo@gmail.com; 6 Facultad de Salud y Ciencias Sociales, Universidad de Las Américas, Santiago 7500975, Chile; 7 Núcleo en Ciencias Ambientales y Alimentarias (NCAA), Universidad de las Américas, Santiago 7500975, Chile

**Keywords:** legumes, intake, season

## Abstract

Background: In Chile, the consumption of legumes at least two times per week is promoted. However, there is a low consumption of legumes. Therefore, our objective is to describe legume consumption in two different seasonal periods. Methods: Serial cross-sectional study: surveys were distributed during summer and winter using different digital platforms. Frequency of consumption, purchase access, and preparation type were investigated. Results: In total, 3280 adults were surveyed in summer and 3339 in winter. The mean age was 33 years. Totals of 97.7% and 97.5% of the population reported consuming legumes in both periods; consumption increased to 3 times per week during winter. In both periods, the main reason for their preference is that they are delicious and nutritious, followed by their use as a meat substitute; the main barriers to their consumption in both periods are that they are expensive (29% in summer and 27.8% in winter) and difficult to prepare. Conclusion: A good consumption of legumes was observed, but with a higher frequency of consumption during winter, with an intake of ≥1 per day; additionally, differences were found in purchases according to season, although no differences were found in the method of preparation.

## 1. Introduction

The eating habits and practices of a population, also known as dietary patterns, can be modified by the environment, the family, or various interacting variables, such as increased purchasing power, access to food products, economic and food globalization, or marketing strategies, among other factors [1]. In Chile, legume consumption is associated with the lower social classes. On the other hand, the consumption of legumes is promoted in the Food-Based Dietary Guidelines (FBDG), which indicate that legumes should be consumed at least twice a week; despite this recommendation, there is a trend that shows a decrease in the intake of legumes in our country [2]. Some results of the National Health Survey (ENS) 2016–2017 mention that 24% of participants—51.4% were women—complied with the recommendations for legume intake. After adjusting for variables, people between 70 and 80 years old, and those living in rural areas and the Maule Region (center of the country), presented a higher probability of compliance [3]. Similarly, another study, this time among students from different universities in Chile, showed that only 23% of the participants complied with the recommendations for legume consumption, with men reporting higher intakes [4].

In this regard, a recent review examined changes in legume consumption during the COVID-19 pandemic. The authors observed maintenance of legume consumption in the eleven studies reviewed from Poland, Denmark, Spain, Lithuania, Turkey, and Zimbabwe. In contrast, other studies from Spain, Italy, and Iran reported increased consumption. Only one study in Brazil registered a decrease [5]. However, during the pandemic in Chile, consumption increased, as indicated by the study by Pye et al., where significant changes were observed in the increase in the percentage of people who consume more servings, specifically from 9.2 to 12% among those who consume 2 to 3 servings/week and 0.2 to 0.3% in those who consume 4–5 servings/week [6]. This situation could be explained because they are non-perishable foods, easy to acquire and store, and have a high yield. In addition, it could be attributed to the fact that they are foods generally consumed in preparations such as hot stews, being a preferred alternative in cold seasons [6]. In this regard, another study confirming the increase in legume consumption in this period showed that 43.5% of women and 38.9% of men met legume recommendations [7]. Finally, other findings in the Chilean population show that low consumption of legumes, associated with a frequency of junk food consumption higher than three times per week, was associated with adverse health outcomes, such as an increase in body weight [8].

On the other hand, as legume production at the local level has decreased significantly in the last 30 years, the Chilean consumption of legumes is quite satisfactory despite this local scenario. In a systematic study on the changes experienced in dietary patterns between 1990 and 2010 conducted in 187 countries, Chile ranked among the top 10 countries consuming pulses, averaging 25–30 grams per day [9]. In addition, the results of the National Food Consumption Survey (ENCA) showed that the average daily intake observed was 17 g/day, with an increase to 21.8 g/day in rural areas, with the most significant consumption in the Central-South Region of the country (25.9 g/day) [10].

In cultural terms, cooking and consuming pulses revitalizes the presence of traditional dishes so that the productive matrix is oriented towards crops whose production responds to the principle of environmental sustainability; an example of this is the implementation of a Mediterranean diet, which promotes, among other things, the replacement of meat with pulses or fish, and entails a significant reduction in the ecological impact, carbon footprint, and water associated with their production [11]. It should be noted that FAO, in turn, has agreed on the need for the economically efficient incorporation of low-cost proteins, such as legumes, which are also positioned as a source of dietary fiber, and play a relevant role as a therapeutic agent in vulnerable populations [12]. In a recent study, food pattern or menu modeling by substituting less healthy dips and spreads with hummus showed that this simple substitution could reduce energy intake, increase protein intake, and more easily facilitate an increase in the legume or vegetable recommendations [13].

In Latin America, limited studies address legume consumption in two different seasonal periods. Therefore, it seems relevant to evaluate dietary aspects related to the consumption and preparation of legumes in Chilean households, in addition to an update of the sociodemographic background, which allows extrapolating variables that influence consumption.

The present study aims to describe the consumption of legumes In two different seasonal periods in Chile.

## 2. Materials and Methods

A serial cross-sectional observational study was conducted in two periods: from 13–30 July 2020 (Winter in Chile), and 3–20 March 2021 (Summer in Chile). Chilean residents of both sexes over 18 years of age were included and people who cannot consume legumes for health reasons were excluded. The survey was disseminated through Google Forms using non-probability sampling and the uncontrolled instrument distribution method; it was distributed through different digital platforms and the social networks Facebook, Instagram, and Twitter. Participants voluntarily participated in the survey and were self-selected for inclusion in the study. To avoid multiple access by the same person, the survey was configured to be applied only once per seasonal period. Once the survey was closed, a total of 3351 surveys were obtained. Of these, 12 records were eliminated because the subjects mentioned that they did not want to participate in the study, so 3339 surveys were included.

The study was conducted in accordance with the Declaration of Helsinki regarding work with human subjects and conformed to the “Singapore Statement on Research Integrity”. It was approved by the Scientific Ethics Committee of the Universidad de Las Américas, Chile, resolution number CEC_FP_2021006.

A structured survey was prepared, previously validated and in digital support, with questions categorized according to the following components: sociodemographic characterization; barriers to legume consumption, such as they are expensive, they are difficult to prepare, they are not liked by the family, they are not fashionable; facilitators such as they are economical, satiating power, they replace meat, they are nutritious, rich, and delicious; the frequency (less than 1 time per week, 1 time per week, 2 times per week, 3 times per week) and the consumption of legumes (including types of legumes: beans, lentils, chickpeas, peas, black beans, soybeans, broad beans, red lentils); season in which they are most consumed (autumn, winter, spring, summer); nutritional contributions or factors associated with their consumption (they provide a large amount of dietary fiber, they are rich in protein, they are high in calories, they have antioxidants, they are protective against chronic diseases, they produce body weight gain, they provide a large amount of fat, they should not be consumed by people with diabetes); forms of preparation (traditional dishes, salads, hamburgers/croquettes; bean paste, mashed pulses, ceviche of pulses, falafel, cream of pulses, bread of flour of pulses, and hummus); and accessibility of purchase (supermarkets, free fairs, online purchase, neighborhood stores, markets). After asking about the consumption of pulses, all those subjects who answered NO were eliminated from the subsequent analyses.

Descriptive statistics were applied to determine the distribution of variables such as consumption, frequency, types of preparation, and accessibility to legumes in the population. The results were presented as relative frequencies and percentages for categorical variables; these categorical variables were compared using Pearson’s chi square test, with a significance of *p* < 0.05. The chi square test was used to compare the two seasons. The analyses were performed using the STATA v.15 statistical packages.

## 3. Results

The sample consisted of 3280 adults of both sexes (75.9% women) in the summer sample and 3339 (87.7% women) in the winter sample, whose ages ranged from 18 to 77 years, with a mean age of 33 years for both seasons. Most of the sample had some higher education (complete or incomplete), and most lived in the Metropolitan Region, Central Region (the most populated region of Chile) (Table 1).

Of the population studied, 97.7% and 97.5% indicated consuming legumes in both periods. Finally, 3205 responses for summer and 3254 for winter were analyzed. Differences were observed in consumption, which increased in the group up to three times per week during the winter. The order of consumer preference for legumes was lentils, beans, chickpeas, and black beans, with similar results in both periods (Table 2). On the other hand, in both periods, the main reason for their preference is that they are delicious and nutritious, followed by their use as a meat substitute and their high yield (i.e., they can feed many people); however, the main barriers to their consumption in both periods are their high economic cost (29% in summer and 27.8% in winter), and that they are difficult to prepare (12.1% in summer and 11.6% in winter). The main ways of preparing them are traditional stews, salad, cream soup, hummus, and hamburger.

Table 3 shows the place of purchase, prices, and knowledge of dietary guidelines. Although the supermarket is the place of choice, it is interesting to note that the place of purchase changes according to the season. During the winter, purchases in supermarkets and Internet purchases increase, but purchases in flea markets and neighborhood stores decrease. During the summer, 60.6% of people indicated that the price of pulses had increased, while in winter this percentage reached 49.7%. Finally, about a third of the subjects mentioned that they were aware of the FBDG recommendations on the consumption of pulses (see Table 3).

## 4. Discussion

The main result observed is that a good consumption of legumes was found, showing an increase in the frequency of consumption during the winter, considering ≥3 times per week; differences were also found in purchases according to the year’s season. However, no differences were found in the way legumes were prepared. In addition, among the factors that promote their consumption are “they are delicious” and “nutritious”, followed by “they substitute meat”. Among the factors that hinder their consumption are their high economic cost and their difficulty in preparation.

Legumes are protein-rich foods that contribute significantly to meeting the protein requirements of the human diet [14,15]. They are also high in fiber, carbohydrates, vitamins, and minerals [16,17]. This information is consistent with our results, which indicated that the main preference for consuming pulses is that they are “delicious and nutritious.” In addition, they are cost-effective. For example, 1 kilogram yields approximately 14–17 servings (traditional stew). A study among adults in Puerto Rico, which aimed to investigate attitudes toward legume consumption and associations with dietary intake, found significant positive associations for the taste and benefits factor, as well as for the social support and cultural beliefs factor with legume intake [18].

On the other hand, the main barriers to their consumption in our study in both seasons are their high economic cost (29% in summer and 27.8% in winter) and their difficulty in preparing (12.1% in summer and 11.6% in winter). Some of the barriers mentioned in other studies are the lack of knowledge about the preparation or cooking of legumes, the perception that legumes are not part of a traditional diet [19], the concern about carbohydrate content [20], or concern about abdominal discomfort or flatulence. In this sense, one study observed that 20% of adult subjects consuming 0.5 cups/day of beans reported increased flatulence after several weeks of pinto bean consumption [21]. Other studies also point out that pulses are less fun, less popular, less suitable for everyday and festive meals, less tasty, less readily available, and more difficult to prepare than meat [22]. Therefore, to increase consumption, it is necessary to make pulses attractive to consumers [23]. However, a meta-analysis, in which 21 randomized controlled clinical trials with isocaloric and/or hypocaloric diets (in both groups) were considered, revealed that people who consumed a daily diet with legumes (132 g/day cooked or one serving per day) obtained a weight reduction of −0.34 kg (*p* < 0.05) at 6 weeks, both in isocaloric and hypocaloric diets [24]. Likewise, another clinical study compared consuming a diet rich in potatoes or legumes (beans). It showed that both were equally effective in reducing insulin resistance and promoting weight loss in people with poor glycemic control [25]. Finally, a recent multicenter study conducted in Latin America analyzed foods associated with body weight gain, and legumes showed no association with weight gain; body weight gain was associated with increased consumption of sugary drinks, pastries, pizza, fried foods, and alcoholic beverages [26].

The main form of preparation observed in our study was the traditional stew, followed by salads, creamy soups, hummus, and burgers. However, a previous analysis comparing according to the type of dietary pattern of the subjects found that those with vegan/vegetarian diets are the highest consumers of legumes and eat a greater variety of types of legumes and show a greater variety of preparations, such as hamburgers, hummus, and salads, that is, recipes that move away from traditional dishes but may be more appealing to the young population [27]. Another study conducted in Latin America, which included university students, showed that individuals following a plant-based diet had the highest consumption of legumes compared to other students following other diets such as western, prudent, etc. [28].

In terms of consumer characteristics, a study characterized frequent purchasers of legumes in the United States using supermarket chains, grocery markets, club stores, big box stores, and Walmart supermarket as a source of information, with military commissary, Nielsen dollar store retail scanner data collected in 2017–2019, and dietary intake from the National Health and Nutrition Examination Survey (NHANES), 2017–2018. It observed an average annual per capita expenditure on pulses of USD 4.76; in addition, there were significant regional differences in the most purchased pulses. In total, 20.5% reported consuming pulses in the past 24 h. Those who consumed pulses were more likely to be Hispanic, with a lower educational level, larger household size, and no differences by age, gender, or income level compared to those who did not consume pulses [29]. Another study from the 2011–2014 NHANES observed that only 5% of subjects consumed pulses daily and that one-third of participants did not consume pulses during the past month. They also observed that the lower price of legumes—especially during the winter months when other vegetables were more expensive—influenced legume consumption and may partly explain why legume consumers increase their consumption during cold seasons. In addition, they note that there is a lack of awareness of the nutritional health benefits of legume consumption, so more educational efforts are needed regarding current dietary recommendations for legumes [30].

In this sense, legume consumption has been positively associated with human health [31] since it plays an active role in the prevention of several diseases, such as cardiovascular disease [32,33], diabetes [34,35], hypertension [36], and colorectal cancer [37]. For example, a meta-analysis found that legume consumption was inversely related to the incidence of ischemic cardiovascular disease, myocardial infarction, and diabetes. Furthermore, consuming 100 g of legumes per week reduced the relative risk of cardiovascular disease by 14% [38]. In the case of diabetes, a randomized clinical trial published in 2019 conducted on university students, in which a legume-based diet was administered to those subjects who received 15 g of the product for 3 months daily for 5 consecutive days and 2 days off, at the end of the intervention showed a reduction in serum levels of glucose, malondialdehyde, and insulin resistance index (HOMA index). The authors indicated that these exciting results could be attributed to polyphenols, such as some isoflavones present in legumes [39]. In the case of hypertension, a meta-analysis analyzing 17 controlled clinical trials suggests that healthy dietary patterns (including legumes) significantly reduced systolic and diastolic blood pressure by 4.2 mm/Hg and 2.3 mm/Hg, respectively [36]. Concerning colorectal cancer, a meta-analysis incorporating cohort studies identified that high consumption of legumes was associated with a lower risk of colorectal cancer [40]. In another meta-analysis of observational studies, the authors concluded that increased legume consumption reduced the risk of adenoma [41]. Legumes also influence declining plasma cholesterol levels. For example, a meta-analysis involving 10 randomized clinical trials comparing legume intake (80–440 g/day) and control diets without legumes identified that a diet incorporating a variety of legumes contributed to an average decrease of −11.7 mg/dL for LDL cholesterol [42].

From an environmental perspective, legume production can contribute to climate change mitigation in several ways (I): they reduce greenhouse gas (GGE) emissions, such as carbon dioxide (CO_2_) and nitrous oxide (N_2_O), compared to farming systems based on mineral fertilization; (II) they play a significant role in soil carbon sequestration; and (III) they induce savings of fossil energy inputs into the system through reduced N [43]. A recent study has shown that increasing legume consumption to two servings per week could slightly improve dietary sustainability when legumes replace meat [44]. In our study, approximately 46% of the subjects consumed legumes ≥2 times per week in both seasons. Other studies conducted in Chile show that 95% of the subjects consumed legumes. However, approximately 40% met the dietary intake recommendations [45]. Although we do not know if their consumption is combined with beef or sausages, the animal protein intake is likely lower in traditional legume preparations (about 30–40 g) than in other preparations without legumes, in which the meat rations can easily increase the protein up to four times (120–160 g). It is important to mention that the sustainability of legumes could be a critical factor in promoting consumption among the young population and favoring domestic production. Finally, the results could contribute to providing valuable information to promote the consumption of this beneficial food product [46] and support strategies aimed at increasing consumption through attractive and innovative preparations and promoting the incorporation of pulses into the Chilean diet, as well as gathering information on the availability and supply chain of pulses in Chile. Currently, due to the events taking place in the world (e.g., the war between Russia and Ukraine), food insecurity is on the rise [47], and legumes can be crucial in the diet of the population, especially the poor, as they are cheap and easily accessible.

Among the strengths of this study, we can mention that we used a previously validated survey that collected data covering the whole country; among the main weaknesses is the fact that it is a cross-sectional study. Therefore, causality cannot be inferred, only association. On the other hand, men respond poorly to dietary surveys. This observed phenomenon could be explained, among other things, by the cultural behavior of women, who are primarily responsible for cooking and buying food. Therefore, they do not feel encouraged to respond to this type of survey. This characteristic has also been observed in other studies published in Latin America [48].

Finally, Chile updated the dietary guidelines for the population. These consist of 10 messages. Message number 3 corresponds to legumes and indicates: “Consume legumes in stews and salads as often as you can,” focusing on the benefits of legume consumption for health and the environment and encouraging the consumption of new preparations without limiting consumption to 2 times per week, as indicated in the previous dietary guide [49].

## 5. Conclusions

Reasonable consumption of legumes was observed, showing an increase in consumption during the winter, with a frequency of consumption of ≥3 times per week; in addition, differences were found in purchases according to the season of the year, although no differences were found in the method of preparation, with the traditional stew the main way of preparing legumes. Additionally, some of the factors that promote consumption are, first, that they are delicious and nutritious, followed by the fact that they are used as a meat substitute. In contrast, some factors that hinder consumption are that they are expensive and difficult to prepare. It is necessary to strengthen nutritional education in the population, as well as to address the nutritional benefits of legumes, promoting innovative preparations that facilitate their consumption in other population groups, incorporating an environmental perspective, since legumes not only provide benefits to human health but also offer benefits to the ecosystem. In this sense, Chile is developing a public–private work to recover traditional legumes and to highlight in the consumer market the nutritional and health benefits of the consumption of legumes in the regular diet. This is achieved through raw materials and food products that allow adding value to legumes to enhance their access to the market and their greater consumption, determine the best form of marketing and participation of each link in the associated value chain, and finally, position the legume issue at the regional and national level.

## Figures and Tables

**Table 1:** Descriptive table. Sociodemographic characteristics of the sample, according to season (summer or winter).

Sociodemographic Characteristics	Distribution of Study Population	
	Summer *n* (%)	Winter *n* (%)
Sex *	3280 (100)	3339 (100)
Female	2489 (75.9)	2927 (87.7)
Male	791 (24.1)	412 (12.3)
Age *		
18–35 years	1519 (46.3)	2012 (60.3)
36–64 years	1603 (48.9)	1276 (38.2)
≥65 years	158 (4.8)	51 (1.5)
Educational level *	Mean (DS)	Mean (DS)
Primary education	17 (0.5)	13 (0.4)
Secondary education	245 (7.5)	202 (6.1)
College/University graduates	2309 (70.4)	2611 (78.2)
College/University dropouts	709 (21.6)	513 (15.4)
Geographical Region		
Southernmost Region	37 (1.1)	38 (1.1)
Central Region	2352 (71.7)	2377 (71.2)
Northern Region	553 (16.9)	552 (16.5)
Southern Region	338 (10.3)	372 (11.1)
People living in the household **		
Two people	700 (21.3)	800 (23.9)
Three people	861 (26.3)	865 (25.9)
Four people	792 (24.2)	836 (25.0)
Five or more people	678 (20.7)	620 (18.6)
Lives alone	249 (7.6)	218 (6.5)

* *p*-value < 0.001; ** *p*-value < 0.05, chi square test.

**Table 2:** Comparison on consumption of pulses, preferences, and barriers to consumption according to season (summer or winter).

Pulses	Distribution of Study Population	
	Summer *n* (%)	Winter ^†^ *n* (%)
Consumption of pulses		
No	75 (2.3)	82 (2.5)
Yes	3205 (97.7)	3254 (97.5)
Frequency of consumption *		
Once a week	1311 (40.9)	1226 (37.7)
Twice a week	1141 (35.6)	1069 (32.8)
Three times a week	349 (10.9)	480 (14.8)
Less than once a week	404 (12.6)	479 (14.7)
Pulses consumed		
Dried peas (Yes)	896 (27.9)	944 (29.0)
Chickpeas (Yes) *	2125 (66.3)	2305 (70.8)
Fava beans (Yes) **	902 (28.1)	1027(31.6)
Lentils (Yes)	3055 (95.3)	3093 (95.1)
Red lentils (Yes) *	289 (9.0)	562 (17.3)
Beans (Yes) *	2821 (88.0)	2726 (83.8)
Soybeans (Yes) **	135 (4.2)	193 (5.9)
Black beans (Yes) **	1440 (44.9)	1667 (51.2)
Red beans (Yes) **	378 (11.8)	474 (14.6)
Preferences		
Inexpensive (Yes)	789 (24.6)	862 (26.5)
Satiating Power (Yes) *	815 (25.4)	973 (29.9)
They replace meat (Yes) *	993 (31.0)	1163 (35.7)
Cost-effective (Yes)	891 (27.8)	967 (29.7)
Delicious and nutritious (Yes)	2915 (91.0)	2958 (90.9)
None of the above (Yes)	101 (3.2)	93 (2.9)
Consumption may be affected by: (beliefs)		
They make you gain weight (Yes)	79 (2.5)	66 (2.0)
They are not trendy (Yes)	195 (6.1)	167 (5.2)
My family does not like them (Yes) **	566 (17.7)	639 (19.6)
They are expensive (Yes) *	929 (29.0)	485 (14.9)
They are difficult to prepare (Yes) *	388 (12.1)	579 (17.8)
None of the above (Yes) *	1390 (43.4)	1587 (48.8)
Preparations		
Ceviche (Yes) *	160 (5.0)	278 (8.5)
Creamy pulses soup (Yes) *	1472 (45.9)	1316 (40.4)
Salad (Yes) *	1688 (52.7)	1881 (57.8)
Falafel (Yes) *	467 (14.6)	616 (18.9)
Burgers/croquettes (Yes) *	955 (29.8)	1248 (38.4)
Hummus (Yes) *	1015 (31.7)	1437 (44.2)
Bread (Yes)	67 (2.0)	57 (1.8)
Black bean paste (Yes)	346 (10.9)	345 (10.6)
Traditional hot-cooked dishes (Yes) *	3144 (98.1)	3134 (96.3)
Pulses puree (Yes) **	677 (21.1)	619 (19.0)

* *p*-value < 0.001; ** *p*-value < 0.05, chi square test; **^†^** missing data (3 for winter).

**Table 3:** Comparison of purchases, prices, and knowledge of dietary guidelines according to season (summer or winter).

Pulses	Distribution of Study Population	
	Summer *n* (%)	Winter *n* (%)
Place of purchase		
Corner store (Yes) **	730 (22.8)	653 (20.1)
Online shopping (Yes) **	396 (12.4)	485 (14.9)
Flea markets (Yes) *	1307 (40.8)	1131 (34.8)
Wholesale market (Yes)	358 (11.2)	339 (10.4)
Supermarkets (Yes) *	2543 (79.3)	2716 (83.5)
Prices *		
Increased	1943 (60.6)	1616 (49.7)
Decreased	20 (0.6)	33 (1.0)
I do not know	668 (20.8)	882 (27.7)
Unchanged	574 (17.9)	723 (22.2)
Knows the FBDG recommendations *		
No	2143 (66.9)	2004 (61.6)
Yes	1062 (33.1)	1250 (38.4)

* *p*-value < 0.001 ** *p*-value < 0.05, chi square test; Food-Based Dietary Guidelines (FBDG).

## Data Availability

The data presented in this study are available on request from the corresponding author. The data are not publicly available due to the data belongs to a project that includes several universities.
